# Spontaneous Oscillatory Rhythms in the Degenerating Mouse Retina Modulate Retinal Ganglion Cell Responses to Electrical Stimulation

**DOI:** 10.3389/fncel.2015.00512

**Published:** 2016-01-12

**Authors:** Yong Sook Goo, Dae Jin Park, Jung Ryul Ahn, Solomon S. Senok

**Affiliations:** ^1^Department of Physiology, Chungbuk National University School of MedicineCheongju, South Korea; ^2^Nano Artificial Vision Research Center, Seoul National University HospitalSeoul, South Korea; ^3^Neuroscience Division, Alfaisal University College of MedicineRiyadh, Saudi Arabia

**Keywords:** retinal degeneration, *rd1* mice, *rd10* mice, retinal ganglion cell, oscillatory local field potential

## Abstract

Characterization of the electrical activity of the retina in the animal models of retinal degeneration has been carried out in part to understand the progression of retinal degenerative diseases like age-related macular degeneration (AMD) and retinitis pigmentosa (RP), but also to determine optimum stimulus paradigms for use with retinal prosthetic devices. The models most studied in this regard have been the two lines of mice deficient in the β-subunit of phosphodiesterase (*rd1* and *rd10* mice), where the degenerating retinas exhibit characteristic spontaneous hyperactivity and oscillatory local field potentials (LFPs). Additionally, there is a robust ~10 Hz rhythmic burst of retinal ganglion cell (RGC) spikes on the trough of the oscillatory LFP. In *rd1* mice, the rhythmic burst of RGC spikes is always phase-locked with the oscillatory LFP and this phase-locking property is preserved regardless of postnatal ages. However, in *rd10* mice, the frequency of the oscillatory rhythm changes according to postnatal age, suggesting that this rhythm might be a marker of the stage of degeneration. Furthermore when a biphasic current stimulus is applied to *rd10* mice degenerate retina, distinct RGC response patterns that correlate with the stage of degeneration emerge. This review also considers the significance of these response properties.

## Robust 10 Hz Oscillatory Rhythm in *rd1* Retina Vs. Fuzzy Oscillatory Rhythm in *rd10* Retina

Spontaneous hyperactivity in ganglion cells (GCs) in degenerating retinas has been reported in several animal strains including *rd1* mice (Margolis et al., 2008; Stasheff, 2008; Borowska et al., 2011; Goo et al., 2011a; Menzler and Zeck, 2011), *rd10* mice (Goo et al., 2011a; Stasheff et al., 2011; Biswas et al., 2014), P23H-1 rats (Sekirnjak et al., 2011), and RCS rats (Pu et al., 2006). This minireview focuses on work carried out using the fast onset retinal degeneration (*rd1*) and slow onset retinal degeneration (*rd10*) mice, since most of our experience has been with these strains.

**Figure 1:**
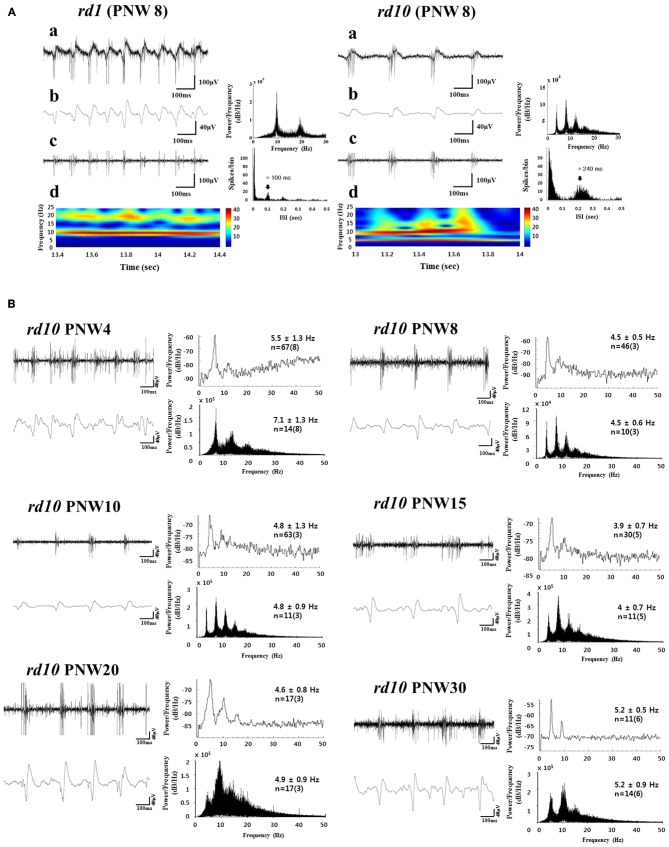
**Spontaneous rhythmic local field potential (LFP) and bursting retinal ganglion cell (RGC) spikes in *rd* retina. (A)** Oscillatory rhythms in retinal degeneration (*rd*) mice models postnatal week (PNW) 8 (*rd1*, left panel; *rd10*, right panel). A typical unfiltered recording of neural activity **(a)**, LFP waveform obtained from low-pass filtering with 20 Hz cutoff frequency and its power spectral density (PSD) estimated by the fast fourier transformation (FFT) **(b)**, and spiking activity obtained by high-pass filtering with 100 Hz cutoff frequency **(c)** shows a temporal structure of rhythmic bursts of spikes, where the inter-burst interval of ~100 ms (corresponding to ~10 Hz) and ~240 ms (corresponding to ~4 Hz), is seen in *rd1* and *rd10* retina respectively. **(d)** The power spectrums through continuous wavelet transform are shown. In *rd1*, hot spot is only observed at ~10 Hz, while in *rd10*, hot spot is more prominent at ~10 Hz but substantial hot spot is also found at ~5 Hz. **(B)** Bursting RGC spikes (left) and its PSD (right) in upper panel, LFP (left) and its PSD (right) in lower panel across different PNW in *rd10* retina. RGC spikes and LFP were obtained with high-pass filtering and low-pass filtering as in **(A)**. The number of cells is indicated, with the number of retinas in parentheses. Except PNW 4, no statistical difference between the spectral peak of bursting RGC spikes and first peak of LFP was found among different age groups (ANOVA, *p* > 0.05). Instead of using Welch method for estimating PSD (Goo et al., 2011a), here we used FFT for PSD estimation, since FFT provides more conspicuous peaks than Welch method. Same data in Goo et al. (2011a) are used. Figures 1A,B are adapted from Figures 3, 5, and 6 from Goo et al. (2011a).

Compared to wild-type (wt) retina, one of the most remarkable features of *rd1* and *rd10* retinas is the presence of spontaneous activity which is characterized by rhythmic bursts of retinal ganglion cell (RGC) spikes superimposed on oscillatory local field potentials (LFPs), otherwise known as the slow wave component (Ye and Goo, 2007; Margolis et al., 2008; Stasheff, 2008; Ryu et al., 2010; Borowska et al., 2011; Goo et al., 2011a; Menzler and Zeck, 2011; Stasheff et al., 2011; Yee et al., 2012; Biswas et al., 2014). In *rd1*, the bursting spikes and the ~10 Hz oscillatory LFPs are always phase-locked regardless of postnatal age (see Figure 1A; Figure 3 in Goo et al., 2011a). In the *rd10* however, oscillatory LFPs occur at a lower frequency (~5 Hz), which is variable according to postnatal age (see Figures 1A,B; Figure 5 in Goo et al., 2011a). Even when there is phase locking between the bursting RGC spikes and the ~5 Hz LFPs in *rd10*, the phase locking is brief and not as robust as in *rd1* (see Figure 1Ad; Figure 6 in Goo et al., 2011a; Figure 3 in Biswas et al., 2014), therefore, the dominant peak of LFPs from postnatal week (PNW) 8 onwards is observed at 10 Hz (rather than 5 Hz) when spectral power is calculated from retinal recording for more than a few minutes (see Figure 1B; Figure 5 in Goo et al., 2011a).

The mechanisms underlying the spontaneous oscillatory rhythm in *rd1* and *rd10* mice have been the subject of considerable study, with the emergence of two mainstream theories. One theory posits that substantial remodeling and rewiring processes upon photoreceptor degeneration transform the retina into a self-signaling neuronal network (Strettoi et al., 2002, 2003; Marc et al., 2003, 2007; Phillips et al., 2010; Jones et al., 2012). While retinal remodeling is a well-recognized sequela of retinal degenerative diseases attended by deafferentation of the neural retina from photoreceptor input, the direct link between such remodeling and the spontaneous oscillatory rhythm has not been established.

The other, now prevailing, theory is that oscillation is an intrinsic property of the electrically-coupled network of AII amacrine and ON cone bipolar cell (BCs; Borowska et al., 2011; Trenholm et al., 2012; Choi et al., 2014; Margolis et al., 2014; Trenholm and Awatramani, 2015). This is supported by the fact that the ~10 Hz oscillation may be induced in wt retina by pharmacological blockade of photoreceptor input to BCs (Trenholm et al., 2012; Trenholm and Awatramani, 2015), or photo-bleaching of the photoreceptors (Menzler et al., 2014). Thus no major rewiring of the retina seems to be required for driving oscillation.

The basic mechanism of oscillation seems to be same in *rd1* and *rd10* retinas. This is supported by pharmacological manipulation of signaling pathways. For instance, block of ionotropic glutamate receptors abolishes oscillations, both in *rd1* (Ye and Goo, 2007; Borowska et al., 2011; Menzler and Zeck, 2011) and *rd10* (Biswas et al., 2014). The gap junction blocker, meclofenamic acid (MFA), abolishes oscillations in both *rd1* (Borowska et al., 2011; Menzler and Zeck, 2011; Trenholm et al., 2012) and *rd10* (Toychiev et al., 2013; Biswas et al., 2014). Block of glycinergic receptors and GABA receptors reduces the frequency of oscillation and increases the LFP amplitude both in *rd1* (Ye and Goo, 2007; Menzler and Zeck, 2011) and in *rd10* (Biswas et al., 2014).

**Figure 2:**
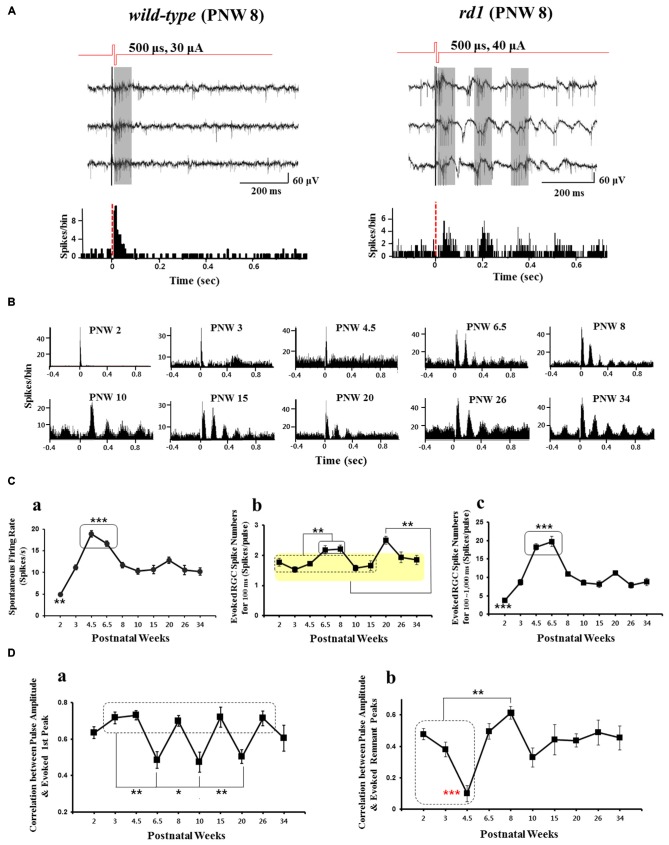
**Electrically-evoked RGC spikes response across different postnatal age. (A)** Typical recording of electrically-evoked RGC spikes in wild-type (left panel), *rd1* (right panel) mice retinas. Biphasic current pulses, 30 and 40 μA amplitude and 500 μs width were applied for wild-type and *rd1*, respectively. Wild-type mice (at postnatal 8 week) typical waveforms show only few evoked spikes with the PSTH derived from 50 stimulus trains showing typical single peak with the latency of less than 100 ms. In *rd1* mice (postnatal 8 week), there is a ~10 Hz background oscillatory rhythm and rhythmic bursting type firing of RGC spikes (marked by gray zone) and multiple (~4) peaks with ~10 Hz rhythm within 400 ms on the PSTH. Here, not the RGC response to voltage stimuli but that to current stimuli is shown. In *rd1* retina, multiple peaks with 10 Hz rhythm in PSTH are observed with current stimulus as in Ye et al. (2008), Ryu et al. (2010), and Goo et al. (2011b). **(B)** Different patterns of PSTH of RGC spikes according to PNW in *rd10* retina. Here, the average spikes per bin of all current amplitudes (5~60 μA) are shown. At PNW 6.5 onwards, typical multiple peaks are observed. **(C)** Number of spontaneous spike and electrically-evoked spike according to PNW in *rd10* retina. See also data used in Park et al. (2015). **(a)** Spontaneous firing rate according to PNW. Asterisk denotes statistical significance (**p < 0.01, ***p < 0.001). **(b)** Electrically-evoked spike number of PSTH first peak (0~100 ms) according to PNW. PNW 6.5, 8 shows higher firing rate than PNW 2 through 15 (**p < 0.01) while PNW 20 shows highest firing rate among all age groups (**p < 0.01). **(c)** Electrically-evoked spike number of PSTH remnant peaks (post-stimulus 100~1000 ms) according to PNW. PNW 2 and PNW 4.5, 6.5 show significantly lower and higher firing rate than all other age groups, respectively (***p < 0.001). **(D)** Correlation between pulse amplitude and evoked spike number of PSTH first peak and remnant peaks according to postnatal age. **(a)** Correlation calculated from PSTH first peak spikes. PNW 6.5, 10, 20 retinas show significantly lower correlation than PNWs shown in the rectangle (PNW 3, 4.5, 8, 15, and 26; **p < 0.01, *p < 0.05). **(b)** Correlation calculated from PSTH remnant peak spikes. While PNW 4.5 retina shows lowest correlation among all age groups (red asterisk, ***p < 0.001), PNW 8 retina shows higher correlation than PNW 2~4.5 (**p < 0.01). Figure 2A is adapted from Figure 1 from Ye et al. (2008). Figures 2B–D are adapted with permission from Figures 1–4 from Park et al. (2015).

In a previous study with *rd10* (Goo et al., 2011a), typical phase-locking between RGC spikes and LFP was not observed at PNW 4, with the frequency of first peak being significantly higher than that in other age groups (ANOVA, *p* < 0.001). The frequency of oscillation becomes stable around 4–5 Hz from PNW 8 onwards, when a strong correlation is also established between the first peak of bursting RGC spikes and oscillatory LFP (see Figure 1B; Figure 5 and Table 1 in Goo et al., 2011a). Recent work with more detailed postnatal follow-up of *rd10* shows that from PNW 2 to 4.5, there is no noticeable oscillation in most retinal patches (see Figure 2B; Figure 4B in Park et al., 2015). Oscillations become a consistent observation in the *rd10* retinal patches from PNW 6.5 (see Figure 2B; Figure 4B in Park et al., 2015). This degeneration-stage dependent frequency change could be explained on the basis of the histological changes of degenerating retinas. At postnatal day (PND) 30~45 (PNW 4.5~6.5), even though there are progressive BC dendritic retraction observed (Gargini et al., 2007), there is still preservation of substantial glutamatergic input from BC to GC. At PNW 8 however, glutamate input to GC is much less (Gargini et al., 2007; Barhoum et al., 2008), resulting in significant decrease in frequency of oscillation in comparison with that at PNW 4 (see Figure 1B: *p* < 0.001 between the LFP at PNW 4 (7.1 ± 1.3 Hz) and PNW8 (4.5 ± 0.6 Hz)).

Changes of the functional properties of BCs occur at early stage of degeneration. Such changes include the aberrant expression of ionotropic glutamate receptors and the loss of expression or reduced activation of metabotropic glutamate receptors on ON-BCs as well as the increase of GABA mediated currents (Varela et al., 2003; Chua et al., 2009; Puthussery et al., 2009; Lin et al., 2012). If these changes exhibit different temporal trajectories in different batches of *rd10*, that could explain some of the differences we found in the *rd10* with regard to when the oscillatory rhythm is first being consistently observed (PNW 8 or PNW 6.5). Although the same strain of *rd10* (B6.CXB1-*Pde6b*^*rd10*^/J), is used in the data of Goo et al. (2011a) and Park et al. (2015), the mice were from different batches.

Since oscillatory rhythm varies with postnatal ages in *rd10* mice, studies have been carried out to see how this might affect the RGC responses to electrical stimulation. If similar degeneration stage dependent electrical properties were to be found in the human retina, stimulation protocols for any retinal prostheses might need to vary according to the stage of the disease.

## Retinal Ganglion Cell Response to Electrical Stimulation According to Postnatal Age

How different types of RGCs respond to electrical stimulation has been studied in many different animals. In primate retina, only ON and OFF parasol cells (comprising ~16% of the RGC population) were found to respond to epiretinal stimulation (Sekirnjak et al., 2008). More recently however, Jepson and collaborators showed that in addition to the ON and OFF parasol cells, ON and OFF midget cells and small bistratified cells (SBCs; which together make up ~75% of primate RGCs), could be activated directly to fire a single spike with submillisecond latency using brief current pulses (Jepson et al., 2013). Since each of the different cell types responded to direct stimulation with similar sensitivity, it is likely that many RGCs within range of a given electrode will respond similarly to a given stimulus, which is desirable for the prosthetic to re-create the appropriate spike pattern regardless of cell types. On the other hand, stimulation that activates RGCs directly is non-physiological and probably difficult for the brain to interpret the neural signal (Im and Fried, 2015).

Jensen and Rizzo (2008) compared response properties of extracellularly recorded RGCs in wt and *rd1* mouse retinas to electrical stimulation. Three types of responses were classified according to spike latencies and number of bursts of spikes (types I, II and III). With the application of glutamate antagonists CNQX and AP-7, electrically evoked activity in RGCs in wt and *rd1* mice was abolished or greatly diminished, suggesting that the RGC responses were indeed network-mediated indirect activation not the direct activation of RGC. Correlation between functional type of RGCs and their electrical responses was however equivocal; in wt, only type II cells, all of which were ON-center RGCs, could be linked to a physiological cell type, while in *rd1* mouse retinas, the only 2 out of 50 RGCs that were responsive to light were of different classes (one was type I, the other type III). Recently, Im and Fried (2015) clearly linked physiologically identified RGC types and their electrical responses to network mediated activation in healthy rabbit retina.

Ever since the elucidation of aberrant spiking activity coupled with oscillatory LFP in *rd* mice (Ye and Goo, 2007; Margolis et al., 2008; Stasheff, 2008; Ryu et al., 2010; Borowska et al., 2011; Goo et al., 2011a; Menzler and Zeck, 2011; Stasheff et al., 2011; Yee et al., 2012; Biswas et al., 2014), the oscillation itself has been regarded as noise which reduces the efficacy of signal transmission within the retinal neuronal network (Yee et al., 2012). When photoreceptor input to voltage-clamped GC is bypassed by direct electrical stimulation of the BC, the evoked synaptic currents show increasing noise levels in *rd1* than age-matched wt mice. This noise level increases with aging to the point of obscuring the evoked response (Figure 9 in Yee et al., 2012). The profound decrease in signal-to-noise ratio in *rd10* retina is such that eliminating the oscillatory activity has been suggested as a treatment strategy for retinal degeneration (Toychiev et al., 2013; Ivanova et al., 2015). Indeed when excessive spiking of RGC is pharmacologically attenuated using a gap junction antagonist, not only is the sensitivity to light increased, but the electrically evoked RGC responses as well (Figures 2, 3 in Toychiev et al., 2013).

With electrical (current or voltage) stimulation, the temporal pattern of RGC responses is very different in wt and *rd1* mice. The post-stimulus time histogram (PSTH) in wt mice typically has a single peak with latency of less than 100 ms. In *rd1* mice at PNW 8 however, the PSTH has multiple peaks (~4 peaks), with ~10 Hz rhythm (in-phase with the LFPs) within 400 ms (see Figure 2A). In *rd10* retina, the emergence of multiple peaks in the PSTH is consistently observed from PNW 6.5 onwards, and the frequency of multiple peaks appears to vary according to postnatal age (see Figure 2B). The response within the first ~100 ms of the post stimulus period is taken as the primary response since the number of evoked spikes were the highest among other subsequent bursts (Ye et al., 2008; Ryu et al., 2010; Goo et al., 2011b). When the temporal epochs of the first three peaks of the PSTH were defined as approximately 0–100 ms, 110–250 ms, and 280–420 ms, the stimulus amplitude modulated RGC response was found to be best represented as expected, within the first peak (see Figure 4 in Ryu et al., 2010). However, on closer examination, the RGC responses in the second peak could also be reliably modulated by pulse amplitude (Figure 4 in Ryu et al., 2010). Additionally, spikes within 0~200 ms were found to be more accurately encoded with pulse amplitude modulation (PAM) than spikes within 0~100 ms alone (Figure 7 in Ryu et al., 2010). These findings support the idea that the first two peaks (rather than just the first peak) should be considered as information-carrying spikes and not just noise.

To address whether oscillation is noise, in this review, we would like to focus on the role of oscillatory rhythm affecting RGC spikes response to electrical stimulus in *rd10* retina. Recent work (Park et al., 2015) examined the potential information-carrying role of the *rd10* PSTH peaks by recording the RGC responses to square-wave current pulses over different postnatal ages (PNW 2~34).

The spontaneous firing rate varies according to postnatal age. It peaks during PNW 4.5–6.5 (*p* < 0.001 in Figure 2Ca) before decreasing rapidly to stabilize at PNW 8 onwards. This is in consonance with a finding of Stasheff et al. (2011) where spontaneous firing rate peaked by PND 50 before decreasing during PND 60–120.

Park et al. (2015) focused on the evoked RGC spike number along the PNW with electrical stimulation, not much considering the stimulus amplitude dependent modulation (Ryu et al., 2010; Goo et al., 2011b) nor the effect of distance between the stimulus channel and recording channel on MEA. By averaging the number of RGC spikes per pulse through all current amplitudes applied (5~60 μA) across all recording channels, it could make the evoked RGC spike number in Figure 2Cb smaller. Nonetheless, statistically significant difference was found along the PNWs. PNW 6.5 and 8 showed higher evoked spikes than age groups of PNW 2 through 15 (**p < 0.01). Similarly, PNW 20 showed higher evoked spikes than all other age groups (**p < 0.01; see Figure 2Cb). When the evoked RGC spikes per pulse for the remnant peaks (post-stimulus 100~1000 ms) were calculated, PNW 4.5 and 6.5 show the highest evoked spikes than other age groups. The evoked RGC spikes of remnant peaks shows great similarity with the spontaneous firing rate (compare Figures 2Ca,Cc). Since the spontaneous spikes are also counted as in evoked spikes in the data of Figure 2, true evoked spikes (post-stimulus spikes minus pre-stimulus spikes) with electrical stimulation should be calculated across age groups to avoid possible contamination of the evoked response by hyperactivity as reported (Yee et al., 2012; Toychiev et al., 2013; Ivanova et al., 2015). Due to possible contamination of spontaneous hyperactivity, it is premature to make conclusions about remnant spikes across age groups.

When the degree of correlation between pulse amplitude and evoked RGC spikes is calculated for first peak and remnant peaks, PAM efficacy was significantly lower at PNW 6.5, 10, and 20 than other age groups (see Figure 2Da). The best correlation (between pulse amplitude and evoked RGC spikes), was at PNW 8, especially for the remnant peaks (see Figure 2Db). Since the correlation curve in Figure 2D shows the mean correlation across all current amplitudes applied (5~60 μA), higher correlation value at PNW 8 implies that the modulation range would be wider like in wt (PNW 2, before degeneration starts) than that in other age groups. As a result, when implementing retinal prosthesis, one might expect better response at PNW 8 than other age groups (Park et al., 2015).

Taking into consideration the fact that best correlation between PAM and evoked RGC spikes is observed especially for the remnant peaks (post 100 ms), than first peak in PNW 8 mice, there is a high possibility of conveying visual information through these remnant peaks at this age. This finding suggests that not only the first peak but also the remnant peaks should be considered as meaningful response. Although no pharmacological dissection of the network-mediated activation of RGCs had been tried in Park et al. (2015), from the second peak all the peaks in PSTH could be regarded as network-mediated activation of RGCs based on the previous studies (see Figures 3, 4 in Jensen and Rizzo, 2008; Ryu et al., 2010; Lee et al., 2013). Besides, correlation between the physiological type of RGCs and their electrical responses like Im and Fried (2015) could help deciding the clinical applicability of network-mediated activation. But first, analysis of the true evoked RGC spikes for remnant peaks and modulation curves of RGC spikes along the current amplitudes applied (5~60 μA) across age groups is needed.

Since all the data in Figure 2 are derived from *in vitro* MEA recording, *in vivo* recording could shed more light on the role of multiple peaks resulting from oscillating LFP. Recording *in vivo* from the superior colliculus (SC), Ivanova and coworkers have reported significantly more spontaneous activity in the light-stimulated *rd10* mouse retina than in age-matched wt mice (see Figure 2 in Ivanova et al., 2015). Due to this aberrant activity in the SC neurons of *rd10* mice, they conclude that RD dysfunction is not limited to the retina but may impair visual activity in higher brain areas as well. Since *in vivo* recording of electrically evoked potentials (EEP) in the *rd10* visual cortex have not been reported, it is not clear whether a corollary of the PSTH multiple peaks seen in the retina will be observed in the visual cortex following electrical stimulation of the retina. This might warrant future studies.

## Summary

1. Both *rd1* and *rd10* degenerate retinas exhibit spontaneous activity consisting of bursting RGC spikes, superimposed and phase-locked with concurrent LFPs. In *rd1*, oscillation occurs at a frequency of 10 Hz, independent of postnatal age while in *rd10* it occurs at 5 Hz, but varies with postnatal age with less robust phase locking. The *rd10* oscillation has the highest frequency at PNW 4, it is stable at 4–5 Hz from PNW 8 onwards.
2. The response to electrical stimulation shows the best correlation between stimulus amplitude and evoked RGC spikes occurring at PNW 8, especially for the remnant peaks (at 100–1000 ms post-stimulus). This is interpreted to mean that the first post- stimulus response peak (100 ms), may not be the only information carrying response of the degenerate retina and that stimulus paradigms for retinal prosthetic devices may need to take into account the stage of retinal degeneration.

## Author Contributions

YSG, DJP, JRA, SSS conceived and designed the experiments; YSG, DJP performed the experiments; DJP, JRA analyzed the data; YSG, DJP, SSS wrote the article.

## Conflict of Interest Statement

The authors declare that the research was conducted in the absence of any commercial or financial relationships that could be construed as a potential conflict of interest.
